# Impact of malaria control interventions on malaria infection and anaemia in low malaria transmission settings: a cross-sectional population-based study in Sudan

**DOI:** 10.1186/s12879-022-07926-x

**Published:** 2022-12-10

**Authors:** Khalid Abdelmutalab Elmardi, Ishag Adam, Elfatih Mohamed Malik, Hmooda Toto Kafy, Mogahid Sheikheldien Abdin, Immo Kleinschmidt, Stef Kremers, Jessica Sophia Gubbels

**Affiliations:** 1 Health Information, Monitoring and Evaluation and Evidence Department, Federal Ministry of Health, Khartoum, Sudan; 2 Department of Health Promotion, Faculty of Health, Medicine and Life Sciences, NUTRIM School of Nutrition and Translational Research in Metabolism, Maastricht, The Netherlands; 3 Department of Obstetrics and Gynecology, Unaizah College of Medicine and Medical Sciences, Qassim University, Unaizah, Saudi Arabia; 4 Faculty of Medicine, University of Khartoum, Khartoum, Sudan; 5 Directorate General of Primary Health Care, Federal Ministry of Health, Khartoum, Sudan; 6 MRC International Statistics and Epidemiology Group, Department of Infectious Disease Epidemiology, London School of Hygiene and Tropical Medicine, London, UK; 7 Faculty of Health Sciences, School of Pathology, Wits Research Institute for Malaria, University of the Witwatersrand, Johannesburg, South Africa; 8 Southern African Development Community Malaria Elimination Eight Secretariat, Windhoek, Namibia

**Keywords:** Impact of malaria interventions, Malaria infection, Malaria control interventions, Anaemia, Low malaria transmission, Sudan

## Abstract

**Background:**

The past two decades were associated with innovation and strengthening of malaria control interventions, which have been increasingly adopted at large scale. Impact evaluations of these interventions were mostly performed in moderate or high malaria transmission areas. This study aimed to evaluate the use and performance of malaria interventions in low transmission areas on malaria infections and anaemia.

**Methods:**

Data from the 2016 Sudan malaria indicator survey was used. Multi-level logistic regression analysis was used to assess the strength of association between real-life community-level utilization of malaria interventions [diagnosis, artemisinin-based combination therapies (ACTs) and long-lasting insecticidal nets (LLINs)] and the study outcomes: malaria infections and anaemia (both overall and moderate-to-severe anaemia).

**Results:**

The study analysis involved 26,469 individuals over 242 clusters. Malaria infection rate was 7.6%, overall anaemia prevalence was 47.5% and moderate-to-severe anaemia prevalence was 4.5%. The average community-level utilization was 31.5% for malaria diagnosis, 29.9% for ACTs and 35.7% for LLINs. The odds of malaria infection was significantly reduced by 14% for each 10% increase in the utilization of malaria diagnosis (adjusted odds ratio (aOR) per 10% utilization 0.86, 95% CI 0.78–0.95, p = 0.004). However, the odds of infection was positively associated with the utilization of LLINs at community-level (aOR per 10% utilization 1.20, 95% CI 1.11–1.29, p < 0.001). No association between malaria infection and utilization of ACTs was identified (aOR per 10% utilization 0.97, 95% CI 0.91–1.04, p = 0.413). None of the interventions was associated with overall anaemia nor moderate-to-severe anaemia.

**Conclusion:**

There was strong evidence that utilization of malaria diagnosis at the community level was highly protective against malaria infection. No protective effect was seen for community utilization of ACTs or LLINs. No association was established between any of the interventions and overall anaemia or moderate-to-severe anaemia. This lack of effectiveness could be due to the low utilization of interventions or the low level of malaria transmission in the study area. Identification and response to barriers of access and low utilization of malaria interventions are crucial. It is crucial to ensure that every suspected malaria case is tested in a timely way, notably in low transmission settings.

**Supplementary Information:**

The online version contains supplementary material available at 10.1186/s12879-022-07926-x.

## Background

During the past two decades, the prevention and treatment of malaria received increased global attention as well as financial support. The fight against malaria in this era witnessed the mass roll-out of three new tools: long-lasting insecticidal nets (LLINs), malaria rapid diagnostic tests (RDTs) and artemisinin-based combination therapies (ACTs) [1]. Prompt malaria case management has a valuable role in malaria control at the community level, with the timely and accurate diagnosis being a cornerstone of effective case management. The development and deployment of RDTs facilitated large scale implementation of the recommendation of confirming malaria cases via parasitological testing prior to treatment [2, 3]. Early and effective treatment of malaria prevents disease progression and reduces transmission at the community level [2]. In addition, insecticide-treated nets, notably LLINs, are recommended by the World Health Organization (WHO) for large scale deployment for populations at risk of malaria in most malaria-endemic settings. LLINs have shown a large public health impact by effectively reducing and preventing infection at the community as well as individual levels [2].

Many researchers studied the impact of malaria interventions and showed their effectiveness using different methodologies, ranging from observational studies to randomized control trials [4–13]. Malaria interventions have a large estimated impact on child health indicators as well, including anaemia [6]. Anaemia can be used as an indicator to assess the progress in malaria control [14, 15], as the strong association between malaria and anaemia has been demonstrated in many endemic countries [8, 16]. Moderate-to-severe anaemia has higher sensitivity than overall anaemia in assessing the burden of malaria [14, 16]. However, utilization of malaria interventions should reach high levels to exhibit measurable impact [17].

Most published impact assessments of malaria interventions were conducted in areas of moderate to high malaria transmission, where reasonably high coverage and/or use was ensured [7–10, 13, 18]. With the global target to reach high levels of interventions utilization and the associated challenges [1, 19, 20], there is a need to know how these interventions impact malaria burden in low transmission settings under real life conditions. This study aimed to assess the use and impact of utilization of malaria control interventions (malaria diagnosis, ACTs and LLINs) by the community, on malaria infection, overall anaemia, and moderate-to-severe anaemia in areas of low malaria transmission in Sudan.

## Methods

### Study setting and interventions

This study was conducted in 12 out of the 18 states in Sudan where LLINs were the predominant malaria control strategy, together with early diagnosis and prompt treatment of malaria cases with ACTs [21]. Nearly half of the malaria burden in the WHO Eastern Mediterranean Region is attributed to Sudan [1], where the prevalence of malaria infection was estimated at 3.3% [22] and the annual malaria cases at around 2 million [1]. Major factors that influence malaria transmission in Sudan include seasonal rains and irrigation schemes, complicated by the fact that Sudan is a low-income country with 32% of its population being urban residents [21, 23, 24]. The predominant malaria parasite is *P. falciparum*; however, an increasing rate of *P. vivax* was recently reported [22, 25, 26]. The main vector is *Anopheles (An.) arabiensis* [27].

At the time of this study, Artesunate plus Sulfadoxine–Pyrimethamine (AS/SP) and Artemether plus Lumefantrine (AL) were the first- and second-line anti-malaria treatments adopted and used in Sudan, respectively. Both malaria microscopy and RDTs are in common use for confirmation of malaria diagnosis in the country. The widely used LLINs during the period of the study were deltamethrin-impregnated nets.

### Study design and sample

**Fig. 1:**
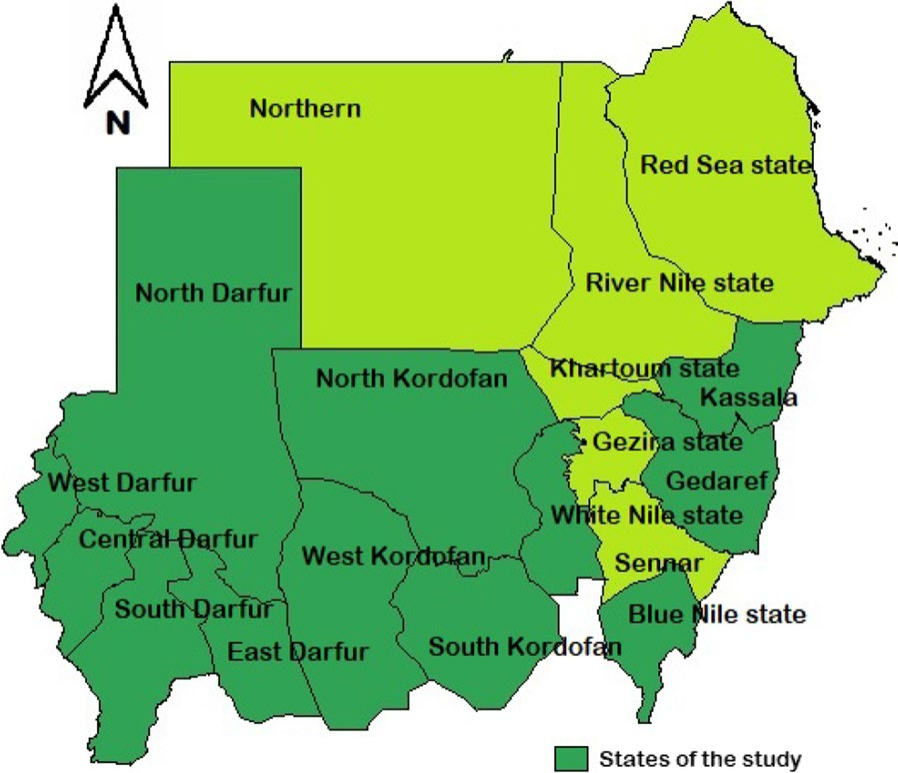
States of the study, Sudan

Data from the 2016 Sudan malaria indicator survey from the states where the interventions (LLINs, malaria diagnosis and treatment with ACTs) were rolled out, were used for this study. These states were Kassala, Gedaref, White Nile, Blue Nile, North Kordofan, South Kordofan, West Kordofan, North Darfur, South Darfur, East Darfur, West Darfur, and Central Darfur (see Fig. 1). The malaria indicator survey is a nationally representative cross-sectional population-based cluster survey. The 2016 survey round was conducted just after the rainy season, in November 2016. The sampling of the survey was designed into two stages. Enumeration areas obtained from the Central Bureau of Statistics were selected randomly with probability proportional to size as the primary sampling units (clusters), stratified by rural/urban status of each area. Then, 20 households were randomly selected from each cluster. Within each household, all members of the household were enrolled in the study. A coded and pretested questionnaire was used as a survey tool to obtain individual and household data [28]. Finger-prick was used to obtain a blood sample from all eligible individuals to assess malaria parasitaemia using malaria rapid diagnostic test (SD BIOLINE Malaria Ag Pf/Pv from STANDARD DIAGNOSTICS INC/SD, South Korea) and haemoglobin level using a field haemoglobin analyser (HemoCue 301 + analyser from Radiometer Group). All methods were carried out in accordance with relevant guidelines and regulations. Written informed consent was obtained from eligible study participants and, for minors, from their parent and/or legal guardian. This study received ethical clearance from the National Ethical and Technical Review Board at the Federal Ministry of Health.

The study questionnaire included socio-demographic characteristics of the study population, whether individuals slept under mosquito nets the night prior to the survey and the type of the net, whether they had fever during the last 14 days, and if yes, whether they were tested parasitologically for malaria; and in case they had a positive result whether they were given an ACT for malaria treatment and when this was taken.

### Outcome variables

The study assessed the strength of association of community-level malaria interventions with three binary outcomes at the individual level separately: malaria infection, overall anaemia, and moderate-to-severe anaemia. The first outcome, malaria infection, is defined as the malaria parasitaemia status of study individuals assessed in all available members of the selected households (regardless of their age) on the survey day through malaria RDT.

Haemoglobin was measured in all children aged 6–59 months and overall anaemia was considered as haemoglobin < 110 g/L [29]. Moderate-to-severe anaemia, as a more sensitive measure of malaria burden [14, 16], was defined as haemoglobin < 80 g/L. Both overall anaemia and moderate-to-severe anaemia were used as study outcomes.

### Independent variables

Community-level utilization of three malaria interventions were the independent variables of interest in this study: (1) malaria diagnosis, (2) ACTs and (3) LLINs. These variables were elicited from the data at the individual level and then aggregated at the cluster level. In this way, independent variables were designed to give an estimation of the community-level utilization of malaria interventions at the cluster level. (To see the individual-level utilization of malaria interventions, refer to Additional file 1: Table S1). Utilization of malaria diagnosis was defined as the cluster level proportion of individuals with fever who were tested for malaria using malaria parasitological test (with microscopy or RDT) in the 14 days prior to the survey date. Similarly, utilization of ACTs was defined as the cluster level proportion of individuals who used ACTs for malaria treatment within 3 days of fever initiation, as a response to parasitologically confirmed malaria. In the same manner, LLINs utilization was defined as the cluster level proportion of individuals who slept under LLIN the night before the survey. These variables were used in the analysis as continuous variables on a percentage scale. Area of residence, classified into rural versus urban, was also used in the analysis to account for its effect.

Age in years (as a continuous variable) and sex (dichotomized into male versus female) were included in the analysis at the individual level. Individual use of LLINs was also included in the analysis.

### Statistics

STATA 16 (Stata Corporation LLC, Texas, USA) was used for the analysis. To describe the overall trend of outcomes and the demographic characteristics of the population, the proportion of the population with the outcome and with the demographic characteristics were calculated as a percentage of the total population assessed. For each malaria intervention, the cluster-level proportion of utilization was estimated and then assigned to every individual in that cluster. Four levels of malaria intervention utilization were defined: low (< 40%), average (40 to < 60%), high (60 to < 80%) and effective (≥ 80%) utilization, as WHO classifies 80% or above as effective community interventions [30].

Considering the two-stage hierarchical nature of this study, a multi-level logistic regression analysis (random-effects model) was used to assess the strength of the association between malaria infection (and overall anaemia as well as moderate-to-severe anaemia) and utilization of malaria interventions, while controlling for potential confounders. This study used individual data (level 1) nested within clusters (the primary sampling units, level 2). As per the multi-level analysis strategy, both clusters and individuals were considered as units of analysis of this study. Based on the objective of the study, the three malaria intervention variables were entered in the multi-level logistic regression analysis. The area of residence (rural/urban) variable was retained in all models because it was used to stratify clusters in the study design. Age and sex variables were included as potential confounders for malaria infection (and overall anaemia as well as moderate-to-severe anaemia) in the multi-level logistic regression analysis, to control for their effects. The cluster was used as the random effect to account for the within-cluster correlation of outcomes.

The strategy for building the multi-level model was based on developing two consecutive models (see strategy for multilevel model building in Additional file 1: Box S1). In the first step, a null model was developed that only included the cluster (higher level) random effect and the intercept. The null model aimed to identify the variation in outcomes that were attributed to cluster variation. In the next step, another model (the full model) was developed that included utilization of malaria intervention variables as well as all potential confounding variables to estimate the strength of the association between the variables and outcomes. Area of residence was retained in full model, to account for the study design effect. Separate models for each outcome (malaria infection, anaemia, and moderate-to-severe anaemia) were built independently.

## Results

In the 242 clusters of the study, a total of 26,469 individuals, distributed in 12 states, were included for the current study analysis. The mean age of the study population was 19.6 years [standard deviation (SD) 18.2 years]. Females represented 57.7% of the study population while about three quarters (76.6%) were rural residents.

### Prevalence of malaria infection and anaemia

Overall, 7.6% [95% confidence interval (CI) 6.5–8.9] of the study sample (N = 26,469) had a malaria infection at the time of the data collection. *P. falciparum* was the major cause of infection with *Plasmodium vivax* representing 9.2%. The mean age of the population with a malaria infection was 13.6 years (95% CI 12.9–14.3). In addition, the malaria infection rate among males was 8.6% compared to 7.0% among females and it was 8.0% among rural compared to 6.4% among urban residents.

Among the 2300 included children between 6 and 59 months old (8.7% of the total study participants), overall anaemia prevalence based on haemoglobin level was 47.5% (95% CI 44.6–50.3). Overall anaemia prevalence was 51.0% among boys compared to 43.9% among girls and it was 48.1% in rural compared to 44.9% in urban communities. The prevalence of moderate-to-severe anaemia among children was 4.5% (95% CI 3.3–6.1). Moderate-to-severe anaemia prevalence was 5.4% among boys compared to 4.1% among girls and it was 4.8% in rural compared to 3.8% in urban communities.

### Cluster-level utilization of malaria interventions

As shown in Table 1, most of the participants were living in areas with either low (< 40%) or average (40 to < 60%) community-level utilization of malaria interventions, while only a minority were living in areas with effective (≥ 80%) utilization. The mean community utilization of malaria interventions was 31.5% for malaria diagnosis, 29.9% for ACTs and 35.7% for LLINs.

**Table 1:** Population distribution by levels of community utilization of malaria control interventions, Sudan 2016

Proportion of the population living in areas with specified community-level utilization of malaria interventions	Level of community utilization of interventions					
	Low (< 40%)	Average (40 to < 60%)	High (60 to < 80%)	Effective (≥ 80%) [30]	Mean (SD)	95% CI of the mean
Malaria diagnosis [in %, Total = 25,877]	68.7%	18.5%	10.4%	2.4%	31.5% (22.3)	29.0–34.0
ACTs [in %, Total = 20,763]	63.5%	21.9%	6.8%	7.9%	29.9% (29.3)	26.2–33.7
LLINs [in %, Total = 24,883]	61.1%	17.7%	13.5%	7.7%	35.7% (25.6)	32.8–38.6

Totals in this table are the total number of individuals living in areas where specified malaria intervention utilization was assessed. Variation in totals is because some clusters (with their study population) were not eligible for specified intervention assessment i.e., no case of fever was reported (malaria diagnosis eligibility) or none was tested positive (ACTs eligibility)

*95% CI* 95% confidence interval, *SD* Standard Deviation, *ACTs* artemisinin-based combination therapies, *LLINs* long-lasting insecticidal nets

### Individual use of LLINs

The average use of LLINs was 33.5% while the potential LLIN coverage (defined as the proportion of households with at least one LLIN per two persons) was 43.8%. The malaria infection rate among individuals who used LLINs was 9.0% and it was 8.4% among non-users (N = 19,901, i.e., only those who were eligible for multi-level multiple logistic regression analysis). Overall anaemia prevalence among children who used LLINs was 47.2% and it was 47.6% among those who did not use it. Similarly, the prevalence of moderate-to-severe anaemia among LLINs users was 4.5% compared to 4.6% among non-users. The multi-level simple logistic regression showed a crude OR (for LLINs users compared to non-users) for malaria infection of 0.81 (95% CI 0.71–0.92, p = 0.001), for overall anaemia of 1.12 (95% CI 0.88–1.43, p = 0.341), and for moderate-to-severe anaemia of 1.16 (95% CI 0.73–1.85, p = 0.537).

(For distribution of outcomes per different levels of community utilization of interventions refer to Additional file 1: Tables S2–S4).

### Association between community-level utilization of malaria interventions and malaria infection

The findings of the multi-level models for the association of malaria interventions and malaria infection are shown in Table 2. The null model demonstrated an intra-class correlation coefficient (ICC) of 0.39 (95% CI 0.33–0.46) indicating that 39% of the variation in malaria infection was attributed to variation in cluster-level characteristics.

The full model showed that, after adjusting for age and sex, the odds of malaria infection was inversely associated with community utilization of diagnosis. The odds of malaria infection was significantly reduced by 14% for each 10% increase in the utilization of malaria diagnosis (adjusted odds ratio (aOR) 0.86, 95% CI 0.78–0.95, p = 0.004). Unexpectedly, the odds of malaria infection was significantly and positively associated with utilization of LLINs and it was increasing with the increasing utilization [aOR (per 10% utilization) 1.20, 95% CI 1.11–1.29, p < 0.001]. The model identified no association between malaria infection and utilization of ACTs (aOR 0.97, 95% CI 0.91–1.04, p = 0.413).

**Table 2:** Association between community-level utilization of malaria interventions and malaria infection, Sudan 2016

Variables	cOR (95% CI)	aOR (95% CI)
Utilization of malaria diagnosis (per 10% utilization)	0.89 (0.80–0.98), p = 0.020	0.86 (0.78–0.95), p = 0.004
Utilization of ACTs (per 10% utilization)	0.98 (0.91–1.05), p = 0.588	0.97 (0.91–1.04), p = 0.413
LLINs utilization (per 10% utilization)	1.17 (1.09–1.26), p < 0.001	1.20 (1.11–1.29), p < 0.001
Area of residence		
Rural (reference)	1	1
Urban	0.82 (0.57–1.19), p = 0.307	0.89 (0.61–1.30), p = 0.555
Sex		
Male (reference)	1	1
Female	0.81 (0.73–0.90), p < 0.001	0.84 (0.75–0.94), p = 0.002
Age (per year)	0.98 (0.97–0.98), p < 0.001	0.98 (0.97–0.98), p < 0.001

Estimates of multi-level (two-level) logistic regression model for the association between community-level utilization of malaria interventions and malaria infection in low transmission area, Sudan 2016.

Intra-class correlation coefficient (ICC) = 0.39 (95% CI 0.33–0.46) [null model]

Total number of observations = 19,901, Total number of clusters (level two units) = 242

*cOR* crude odds ratio, *95% CI* 95% confidence interval, *aOR (for the final model)* adjusted odds ratio, *ACTs* artemisinin-based combination therapies, *LLINs* long-lasting insecticidal nets

### Association between community-level utilization of malaria interventions and overall and moderate-to-severe anaemia

As shown in Table 3, none of the malaria interventions demonstrated a significant association with neither overall anaemia or moderate-to-severe anaemia in the bivariate multi-level regression analysis. Thus, no further models were developed.

**Table 3:** Association between community-level utilization of malaria interventions and overall anaemia and moderate-to-severe anaemia, Sudan 2016

Variables	Overall anaemia cOR (95% CI)	Moderate-to-severe anaemia cOR (95% CI)
Utilization of malaria diagnosis (per 10% utilization)	1.00 (0.93–1.07), p = 0.988	0.94 (0.83–1.06), p = 0.303
Utilization of ACTs (per 10% utilization)	1.01 (0.96–1.0), p = 0.780	1.01 (0.94–1.098), p = 0.714
LLINs utilization (per 10% utilization)	0.98 (0.93–1.03), p = 0.415	1.00 (0.91–1.09), p = 0.983
Area of residence		
Rural (reference)	1	1
Urban	0.88 (0.64–1.21), p = 0.434	0.93 (0.54–1.61), p = 0.794
Sex		
Male (reference)	1	1
Female	0.72 (0.58–0.89), p = 0.002	0.55 (0.34–0.89), p = 0.016
Age (per year)	0.66 (0.60–0.73), p < 0.001	0.88 (0.71–1.08), p = 0.217

Estimates of multi-level (two-level) logistic regression for the association between community-level utilization of malaria interventions and anaemia (and moderate-to-severe) in low transmission area, Sudan 2016

Intra-class correlation coefficient (ICC) = 0.12 (95% CI 0.07–0.19) [null model]

Total number of observations = 1550, Total number of clusters (level two units) = 222

*cOR* crude odds ratio, *95% CI* 95% confidence interval, *ACTs* artemisinin-based combination therapies, *LLINs* long-lasting insecticidal nets

## Discussion

This study aimed to assess the community-level utilization of several malaria control interventions and their impact on malaria infection, overall anaemia, and moderate-to-severe anaemia, in a population living in areas of low malaria transmission in Sudan. The study identified that malaria infection rates were relatively low (7.6%). The peak of malaria infections in this study was among the adolescent population. This low level of malaria infection and the age group most at risk that was concentrated in older children in this study are typical findings for low-malaria transmission areas, while in high transmission areas higher infection rates, morbidity, and mortality cluster among younger children [3].

The majority of the population live in areas where the community-level utilization of each of the three malaria interventions was low. The average utilization of interventions hardly exceeded a third of the population. Individual use of LLINs was also low, with only one-third reporting that they had slept under LLINs the night prior to the survey. However, this study did not identify the reasons for the low usage of the intervention, and whether these related to supply or demand. It would be short-sighted to simply blame the population for the low utilization of malaria interventions identified by this study, since potential coverage with one LLINs per two household members was also low at 43.8%, indicating insufficient provision. Evidence from other studies showed that low universal coverage of LLINs was the major cause of low utilization by the population [31–34]. This low utilization of interventions hinders effective control and reduction of malaria, for which a target of ≥ 80% has been set by the WHO [30]. Worldwide, the low utilization of malaria interventions is indicated as a major challenge [1, 19, 20]. With the current trend, the WHO does not expect to achieve the 2030 global malaria technical strategy (GTS) and the sustainable development goals (SDGs) targets for malaria morbidity and mortality reduction. It was estimated that, if the current trend continues, the global incidence of malaria could be off track of the GTS by 87% by 2030 [1]. The need to identify and respond to the obstacles preventing access to and effective utilization of the current interventions remains crucial.

This study found that the odds of malaria infection decreased with the increasing utilization of malaria diagnosis in the community. This indicates that there was a protective effect of increasing access and community use of diagnosis against malaria infection: the more participants received malaria diagnostic services on time, the lower the risk of people within the community getting malaria. This is logical, especially if the diagnosis is followed by prompt effective use of antimalaria treatment, thus offering an opportunity to early elicit cases and clear gametocytes, which in turn reduces transmission [3]. The quantified protective relationship of malaria diagnosis and infection identified in this study confirms the need for enhancing access and utilization to this key case-management tool.

By contrast, utilization of ACTs was not significantly associated with malaria infection in this study. This could probably be due to the low community-level utilization of the ACT. Furthermore, it is important to mention that a decreasing efficacy of AS/SP was reported from Sudan, which was mainly attributed to the failing SP component [35]. This low AS/SP efficacy could have played a role in the current lack of effect, too. Modelling studies showed that a larger impact of case management, notably ACTs, in reducing malaria burden was noticed when coverage was high, in which the coverage-effectiveness curve slope non-linearly increases with increasing in coverage [36–38]. Findings from this study support these mathematical models’ findings, which conclusively denotes that in low transmission areas low community utilization of ACTs may have little or no effect and sufficiently high usage is crucial. The impact of ACTs alone in areas with a prior low transmission state demonstrated some effect in modelling studies, with little effect perceived in areas with prior high transmission [39].

On the other hand, the odds of malaria infection in this study increased with an increasing rate of utilization of LLINs. In other words, the effectiveness of LLINs on malaria infection was not evident at this low level of community utilization. This may be because the impact of community interventions is more prominent with high coverage [17]. In a controlled study in Côte d’Ivoire, high LLINs coverage (95–100%) was associated with a decrease in malaria transmission [18]. In mathematical models, the pattern of malaria prevalence was found consistent with the trend in increasing LLINs coverage [37, 39]. However, coverage alone is not enough to demonstrate the effectiveness, unless combined with community utilization [40]. It is not clear whether the lack of the impact of the community-level utilization of LLINs in this study on malaria infection was actually due to the low community utilization only, or also due to other factors. For instance, insecticide resistance could have played a role; vector (*An. arabiensis*) resistance to Deltamethrin was detected in Sudan in many studies [27, 41]. Moreover, decreasing mosquito mortality was noticed associated with an increasing trend in LLINs use in Sudan [27, 41]. However, it is less likely to attribute the lack of impact of LLINs in this study to the insecticide resistance, since previous evidence, including from Sudan, showed that even in the face of insecticide resistance, LLINs are still effective in providing protection against malaria [42]. The role of change in biting behaviors of *An. arabiensis* in explaining the unexpected association observed for community utilization of LLINs and the outcome was not studied. However, change in vector behaviors, if present, could not be ignored. The positive association between community-level utilization of LLINs and infection might be the result of the reverse causation where higher utilization of and probably coverage with LLINs were driven by higher burden. In other words, this might reflect that higher mosquito numbers drive the use of nets to diminish the nuisance of mosquito bites.

Nonetheless, this study showed that sleeping under LLINs has an individual protective effect against malaria infection. In modelling studies, the effectiveness of LLINs use was demonstrated in terms of reducing incidence and transmission [43]. An association of low mortality was observed with individuals using ITNs compared to non-users [44]. In a systematic review study, the effect of individual net use was found protective, but not the household use [45]. The individual protective effect of LLIN use seen in this study could either be due to the mechanical barrier of nets or the insecticidal effect, or, more probably, both [46, 47].

This study showed that almost half (47.5%) of the children under five were anaemic, with 4.5% suffering from moderate-to-severe anaemia. The study did not find an association between overall anaemia and community-level utilization of malaria interventions. Moderate to severe anaemia, which is a more sensitive indicator to measure malaria burden [14, 16], similarly did not show an association with malaria interventions. By contrast, findings from other studies did demonstrate a link between malaria interventions and anaemia [7–10]. The absence of association between malaria interventions and anaemia seen in the current study could be explained either by the low community utilization of interventions, or the low malaria transmission level seen in the study area. It is expected that in low transmission settings, chronic malaria and repeated malaria infections are less frequent and hence may not be major contributors to anaemia in such settings [15, 48, 49]. In low to moderate transmission settings, including from Sudan, anaemia was previously found to be associated with malaria infection, but not with the transmission level [15, 28, 48–50].

When evaluating progress in malaria control, the way we are assessing the impact of malaria interventions needs some fine-tuning [6, 51, 52]. As a lesson learnt from this study, two important factors, among others, should be considered while measuring the impact of malaria interventions: the utilization level of interventions and the prior level of malaria transmission [36–39, 43, 51]. The impact of malaria interventions was noticed to vary with the level of transmission [36, 39, 43, 51].

This study did not assess the efficacy of malaria interventions in the ideal situation where high coverage and proper use were ensured or in areas of moderate and/or high malaria transmission. It is among the few studies that assessed the impact of the population use of malaria diagnosis, as a case-management tool, on malaria burden. Furthermore, the study used a multi-level analysis strategy, considering both clusters and individuals as units of this study, while adjusting for potential confounders. The large sample size of this study is one of its strengths. One of the limitations of this study is that it used individual use of LLIN the night prior to the survey as a proxy indicator for measuring utilization. This in fact does not reflect the real exposure–effect relationship but is commonly used as standard indicator in malaria indicator surveys. Other limitation of this study is that it did not include larval source management (LSM) activities in urban settings in the evaluation. This was due to the technical and operational difficulties in assessing this intervention. Evidence from a systematic review showed that proximity to breeding sites is associated with a higher risk of malaria [45].

## Conclusion

The findings of this study showed that malaria prevalence in the study area was relatively low (7.6%). Although nearly half of the children under 5 years were anaemic, few had moderate-to-severe anaemia. Utilization of malaria interventions was low, and the majority of the population were living in areas where intervention utilization was classified as less effective. Utilization of malaria diagnosis demonstrated as a significant protective effect against malaria infection, but this effect was not seen with ACTs or LLINs utilization. None of the interventions showed an association with overall anaemia or moderate-to-severe anaemia. The lack of effect of ACTs and LLINs could potentially be attributed to the low community utilization of these interventions or the prior low level of malaria transmission in the study area. The current findings indicate that national malaria control programs and donor agencies have to prioritize high interventions access as well as utilization.

## Supplementary Information

**Additionalfile 1: Box S1.** Strategy for multilevel model building. **Table S1.** Individual-level utilization of malaria interventions. **Table S2.** Malaria infection rate by malaria interventions and by community-level utilization of malaria interventions. **Table S3.** Prevalence of anaemia (overall) by malaria interventions and by community-level utilizationof malaria interventions. **Table S4.** Prevalence of moderate-to-severe anaemia by malaria interventions and by community-level utilization of malaria interventions.

## Data Availability

The data that support the findings of this study are available from Sudan Federal Ministry of Health, but restrictions apply to the availability of these data, which were used under license for the current study, and so are not publicly available. Data are however available from the authors upon reasonable request and with permission of Sudan Federal Ministry of Health.

## Abbreviations

ACT: Artemisinin-based combination therapies
AL: Artemether plus lumefantrine
*An. arabiensis*: *Anopheles arabiensis*
aOR: Adjusted odds ratio
AS/SP: Artesunate plus sulfadoxine–pyrimethamine
cOR: Crude odds ratio
GTS: Global malaria technical strategy
ICC: Intra-class correlation coefficient
IRS: Indoor residual spraying
ITNs: Insecticide-treated bed-nets
LLINs: Long-lasting insecticidal nets
LSM: Larval source management
*P. falciparum*: *Plasmodium falciparum*
RDTs: Malaria rapid diagnostic tests
SD: Standard deviation
SDGs: Sustainable development goals
WHO: World Health Organization
95% CI: 95% confidence interval

## References

[1] World Health Organization (2020). World malaria report 2020: 20 years of global progress and challenges.

[2] World Health Organization (2021). WHO guidelines for malaria.

[3] World Health Organization (2015). Guidelines for the treatment of malaria.

[4] Steketee RW, Campbell CC (2010). Impact of national malaria control scale-up programmes in Africa: magnitude and attribution of effects. Malar J.

[5] Thomson MC, Ukawuba I, Hershey CL, Bennett A, Ceccato P, Lyon B (2017). Using rainfall and temperature data in the evaluation of national malaria control programs in Africa. Am J Trop Med Hyg.

[6] Gansey RJ (2020). Role of malaria control in improving child health in mainland Tanzania: evidence from a rapid policy scale-up. World Dev.

[7] Hershey CL, Florey LS, Ali D, Bennett A, Luhanga M, Mathanga DP (2017). Malaria control interventions contributed to declines in malaria parasitemia, severe anemia, and all-cause mortality in children less than 5 years of age in Malawi, 2000–2010. Am J Trop Med Hyg.

[8] Eckert E, Florey LS, Tongren JE, Salgado SR, Rukundo A, Habimana JP (2017). Impact evaluation of malaria control interventions on morbidity and all-cause child mortality in Rwanda, 2000–2010. Am J Trop Med Hyg.

[9] Aregawi MW, Ali AS, Al-mafazy A, Molteni F, Katikiti S, Warsame M (2011). Reductions in malaria and anaemia case and death burden at hospitals following scale-up of malaria control in Zanzibar, 1999–2008. Malar J.

[10] Smithson P, Florey L, Salgado SR, Hershey CL, Masanja H, Bhattarai A (2015). Impact of malaria control on mortality and anemia among Tanzanian children less than five years of age, 1999–2010. PLoS ONE.

[11] Gimba B, Bala SI (2017). Modeling the impact of bed-net use and treatment on malaria transmission dynamics. Int Sch Res Not.

[12] Sagara I, Fofana B, Gaudart J, Sidibe B, Togo A, Toure S (2012). Repeated artemisinin-based combination therapies in a malaria hyperendemic area of Mali: efficacy, safety, and public health impact. Am J Trop Med Hyg.

[13] Bhattarai A, Ali AS, Kachur SP, Mårtensson A, Abbas AK, Khatib R (2007). Impact of artemisinin-based combination therapy and insecticide-treated nets on malaria burden in Zanzibar. PLoS Med.

[14] Mathanga DP, Campbell CH, Eng J, Wolkon A, Bronzan RN, Malenga GJ (2010). Comparison of anaemia and parasitaemia as indicators of malaria control in household and EPI-health facility surveys in Malawi. Malar J.

[15] Castelli F, Sulis G, Caligaris S (2014). The relationship between anaemia and malaria: apparently simple, yet controversial. Trans R Soc Trop Med Hyg.

[16] White NJ (2018). Anaemia and malaria. Malar J.

[17] Mbah MLN, Parikh S, Galvani AP (2015). Comparing the impact of artemisinin-based combination therapies on malaria transmission in sub-Saharan Africa. Am J Trop Med Hyg.

[18] Ouattara AF, Dagnogo M, Constant EA, Koné M, Raso G, Tanner M (2014). Transmission of malaria in relation to distribution and coverage of long-lasting insecticidal nets in central Côte d’Ivoire. Malar J.

[19] Runge M, Snow RW, Molteni F, Thawer S, Mohamed A, Mandike R (2020). Simulating the council-specific impact of anti-malaria interventions: a tool to support malaria strategic planning in Tanzania. PLoS ONE.

[20] Griffin JT, Hollingsworth TD, Okell LC, Churcher TS, White M, Hinsley W (2010). Reducing *Plasmodium falciparum* malaria transmission in Africa: a model-based evaluation of intervention strategies. PLoS Med.

[21] Noor AM, ElMardi KA, Abdelgader TM, Patil AP, Amine AAA, Bakhiet S (2012). Malaria risk mapping for control in the Republic of Sudan. Am J Trop Med Hyg.

[22] Federal Ministry of Health. Malaria indicator survey 2012 in the Republic of Sudan. Khartoum, Sudan; 2013.

[23] Central Bureau of Statistics. Sudan population data sheet 2018. Sudan, Khartoum; 2018.

[24] Federal Ministry of Health (2011). National health sector strategic plan (2012–2016).

[25] Suliman MMA, Hamad BM, Albasheer MMA, Elhadi M, Amin Mustafa M, Elobied M (2016). Molecular evidence of high proportion of *Plasmodium vivax* malaria infection in White Nile Area in Sudan. J Parasitol Res.

[26] Elgoraish AG, Elzaki SEG, Ahmed RT, Ahmed AI, Fadlalmula HA, Abdalgader Mohamed S (2019). Epidemiology and distribution of *Plasmodium vivax* malaria in Sudan. Trans R Soc Trop Med Hyg.

[27] Ismail BA, Kafy HT, Sulieman JE, Subramaniam K, Thomas B, Mnzava A (2018). Temporal and spatial trends in insecticide resistance in *Anopheles arabiensis* in Sudan: outcomes from an evaluation of implications of insecticide resistance for malaria vector control. Parasites Vectors.

[28] Elmardi KA, Adam I, Malik EM, Ibrahim AA, Elhassan AH, Kafy HT (2020). Anaemia prevalence and determinants in under 5 years children: findings of a cross-sectional population-based study in Sudan. BMC Pediatr.

[29] World Health Organization (2011). Haemoglobin concentrations for the diagnosis of anaemia and assessment of severity.

[30] World Health Organization (2021). Global technical strategy for malaria 2016–2030. 2021 update.

[31] Solomon T, Loha E, Deressa W, Gari T, Overgaard HJ, Lindtjørn B (2019). Low use of long-lasting insecticidal nets for malaria prevention in south-central Ethiopia: a community-based cohort study. PLoS ONE.

[32] Gobena T, Berhane Y, Worku A (2012). Low long-lasting insecticide nets (LLINs) use among household members for protection against mosquito bite in kersa, Eastern Ethiopia. BMC Public Health.

[33] Raghavendra K, Chourasia MK, Swain DK, Bhatt RM, Uragayala S, Dutta GDP (2017). Monitoring of long-lasting insecticidal nets (LLINs) coverage versus utilization: a community-based survey in malaria endemic villages of Central India. Malar J.

[34] Khanam F, Hossain MB, Chowdhury TR, Rahman MS, Kabir M, Naher S (2018). Exploring the gap between coverage, access, and utilization of long-lasting insecticide-treated nets (LLINs) among the households of malaria endemic districts in Bangladesh. Malar J.

[35] Adeel AA, Elnour FAA, Elmardi KA, Abd-Elmajid MB, Elhelo MM, Ali MS (2016). High efficacy of artemether–lumefantrine and declining efficacy of artesunate + sulfadoxine–pyrimethamine against *Plasmodium falciparum* in Sudan (2010–2015): evidence from in vivo and molecular marker studies. Malar J.

[36] Ross A, Tediosi F, Smith T, Utzinger J, Maire N, Tanner M (2006). An approach to model the costs and effects of case management of *Plasmodium falciparum* malaria in sub-Saharan Africa. Am J Trop Med Hyg.

[37] Bhatt S, Weiss DJ, Cameron E, Bisanzio D, Mappin B, Dalrymple U (2015). The effect of malaria control on *Plasmodium falciparum* in Africa between 2000 and 2015. Nature.

[38] Briët OJT, Impoinvil DE, Chitnis N, Pothin E, Lemoine JF, Frederic J (2019). Models of effectiveness of interventions against malaria transmitted by *Anopheles albimanus*. Malar J.

[39] Briët OJ, Penny MA (2013). Repeated mass distributions and continuous distribution of long-lasting insecticidal nets: modelling sustainability of health benefits from mosquito nets, depending on case management. Malar J.

[40] Awine T, Silal SP (2020). Accounting for regional transmission variability and the impact of malaria control interventions in Ghana: a population level mathematical modelling approach. Malar J.

[41] Implications of Insecticide Resistance Consortium (2018). Implications of insecticide resistance for malaria vector control with long-lasting insecticidal nets: trends in pyrethroid resistance during a WHO-coordinated multi-country prospective study. Parasites Vectors.

[42] Kleinschmidt I, Bradley J, Knox TB, Mnzava AP, Kafy HT, Mbogo C (2018). Implications of insecticide resistance for malaria vector control with long-lasting insecticidal nets: a WHO-coordinated, prospective, international, observational cohort study. Lancet Infect Dis.

[43] Mukhtar AYA, Munyakazi JB, Ouifki R, Clark AE (2018). Modelling the effect of bednet coverage on malaria transmission in South Sudan. PLoS ONE.

[44] Rumisha SF, Smith TA, Masanja H, Abdulla S, Vounatsou P (2014). Relationship between child survival and malaria transmission: an analysis of the malaria transmission intensity and mortality burden across Africa (MTIMBA) project data in Rufiji demographic surveillance system, Tanzania. Malar J.

[45] Bannister-Tyrrell M, Verdonck K, Hausmann-Muela S, Gryseels C, Muela Ribera J, Peeters Grietens K (2017). Defining micro-epidemiology for malaria elimination: systematic review and meta-analysis. Malar J.

[46] Centers for Disease Control and Prevention. Malaria—malaria worldwide—How can malaria cases and deaths be reduced? Insecticide-treated bed nets. Centers for Disease Control and Prevention. https://www.cdc.gov/malaria/malaria_worldwide/reduction/itn.html. Accessed 27 Dec 2021.

[47] Krezanoski P (2016). Delivering insecticide-treated nets for malaria prevention: innovative strategies. Res Rep Trop Med.

[48] Mensah-Brown HE, Abugri J, Asante KP, Dwomoh D, Dosoo D, Atuguba F (2017). Assessing the impact of differences in malaria transmission intensity on clinical and haematological indices in children with malaria. Malar J.

[49] Kilama M, Lindsay SW, Greenhouse B, Arinaitwe E, Kamya MR, Staedke SG (2015). Malaria transmission, infection, and disease at three sites with varied transmission intensity in Uganda: implications for malaria control. Am J Trop Med Hyg.

[50] Ntenda PAM, Chilumpha S, Mwenyenkulu ET, Kazambwe JF, El-Meidany W (2019). Clinical malaria and the potential risk of anaemia among preschool-aged children: a population-based study of the 2015–2016 Malawi micronutrient survey. Infect Dis Poverty.

[51] Hay SI, Smith DL, Snow RW (2008). Measuring malaria endemicity from intense to interrupted transmission. Lancet Infect Dis.

[52] Rowe AK (2017). Assessing the health impact of malaria control interventions in the MDG/sustainable development goal era: a new generation of impact evaluations. Am J Trop Med Hyg.

