# Supplementary material for: Impact of malaria control interventions on malaria infection and anaemia in low malaria transmission settings: a cross-sectional population-based study in Sudan
Source: BMC Infect Dis. 2022 Dec 10;22:927. doi: 10.1186/s12879-022-07926-x (PMC9737986; doi:10.1186/s12879-022-07926-x)
Supplement: Supplementary file 1 — Additionalfile 1: Box S1. Strategy for multilevel model building. Table S1. Individual-level utilization of malaria interventions. Table S2. Malaria infection rate by malaria interventions and by community-level utilization of malaria interventions. Table S3. Prevalence of anaemia (overall) by malaria interventions and by community-level utilizationof malaria interventions. Table S4. Prevalence of moderate-to-severe anaemia by malaria interventions and by community-level utilization of malaria interventions. [file 12879_2022_7926_MOESM1_ESM.docx]

**Additional file 1**

**Box S1: Strategy for multilevel model building**

| **Models** | **The null model** | **The full model** |
| --- | --- | --- |
| **Variables added to previous model** | No predictors were included. Only the higher level (cluster) random effect and the intercept | Malaria interventions (utilization of malaria diagnosis, utilization of ACTs and utilization of LLINs). Area of residence was also retained in this model because it was used for the study design (stratification). All these are higher-level variables.  Other relevant level-one variables (age and sex) were also included. |
| **Model objective** | To identify the variation in malaria infection (and anaemia and moderate-to-severe anaemia) that is attributable to the higher level (cluster) | To estimate the strength of the relationship between interventions and study outcomes while controlling for potential confounding factors |

**Table S1:** **Individual-level utilization of malaria interventions**

| **Malaria interventions** | **Proportion of individuals using interventions** | |
| --- | --- | --- |
|  | **Percent** | **Number of clusters involved** |
| **Malaria diagnosis** [Total=3579] | 46.8% | 511 |
| **ACTs** [Total=1017] | 36.4% | 504 |
| **LLINs** [Total=26469] | 33.5% | 544 |
| *ACTs: artemisinin-based combination therapies. LLINs: long-lasting insecticidal nets.*  *Totals in this table are the total number of individuals who were assessed for use of each malaria intervention.* | | |

**Table S2: Malaria infection rate by malaria interventions and by community-level utilization of malaria interventions**

| **Malaria infection rate** | **Malaria interventions** | **Community-level utilization of malaria interventions** | | | | |
| --- | --- | --- | --- | --- | --- | --- |
|  |  | **Low (<40%)** | **Average (40-<60%)** | **High (60-<80%)** | **Effective (≥80%)** | **Overall** |
| **Proportion of the population with malaria infection**  [% (Total)] | **Malaria diagnosis**  [Grand total=25877] | 8.6% (17776) | 6.0% (4788) | 5.1% (2696) | 8.9% (617) | 7.8% (25877) |
|  | **ACTs**  [Grand total=20763] | 8.3% (13175) | 9.3% (4544) | 8.9% (1402) | 7.6% (1642) | 8.5% (20763) |
|  | **LLINs**  [Grand total=24883] | 6.7% (15204) | 7.9% (4406) | 10.6% (3349) | 12.0% (1924) | 7.9% (24883) |
| *ACTs: artemisinin-based combination therapies. LLINs: long-lasting insecticidal nets.*  *Grand totals in this table are the total number of individuals for which malaria infection prevalence was calculated among those living in specified level of malaria intervention utilization.* | | | | | | |

**Table S3: Prevalence of anaemia (overall) by malaria interventions and by community-level utilization of malaria interventions**

| **Prevalence of anaemia** | **Malaria interventions** | **Community-level utilization of malaria interventions** | | | | |
| --- | --- | --- | --- | --- | --- | --- |
|  |  | **Low (<40%)** | **Average (40-<60%)** | **High (60-<80%)** | **Effective (≥80%)** | **Overall** |
| **Proportion of the population with anaemia** [% (Total)] | **Malaria diagnosis**  [Grand total=2008] | 47.8% (1404) | 46.0% (374) | 49.5% (200) | 30.0% (30) | 47.4% (2008) |
|  | **ACTs**  [Grand total=1631] | 46.3% (1049) | 53.0% (353) | 42.9% (105) | 46.8% (124) | 47.6% (1631) |
|  | **LLINs**  [Grand total=1916] | 48.7% (1184) | 44.6% (325) | 40.4% (272) | 48.9% (135) | 46.9% (1916) |
| *ACTs: artemisinin-based combination therapies. LLINs: long-lasting insecticidal nets.*  *Grand totals in this table are the total number of under 5 children for which anaemia prevalence was calculated among those living in specified level of malaria intervention utilization.* | | | | | | |

**Table S4: Prevalence of moderate-to-severe anaemia by malaria interventions and by community-level utilization of malaria interventions**

| **Prevalence of moderate-to-severe anaemia** | **Malaria interventions** | **Community-level utilization of malaria interventions** | | | | |
| --- | --- | --- | --- | --- | --- | --- |
|  |  | **Low (<40%)** | **Average (40-<60%)** | **High (60-<80%)** | **Effective (≥80%)** | **Overall** |
| **Proportion of the population with moderate-to-severe anaemia** [% (Total)] | **Malaria diagnosis**  [Grand total=2008] | 5.2% (1404) | 3.2% (374) | 3.5% (200) | 3.3% (30) | 4.6% (2008) |
|  | **ACTs**  [Grand total=1631] | 4.3% (1049) | 8.2% (353) | 3.8% (105) | 3.2% (124) | 5.0% (1631) |
|  | **LLINs**  [Grand total=1916] | 4.2% (1184) | 4.6% (325) | 2.2% (272) | 5.9% (135) | 4.1% (1916) |
| *ACTs: artemisinin-based combination therapies. LLINs: long-lasting insecticidal nets.*  *Grand totals in this table are the total number of under 5 children for which moderate-to-severe anaemia prevalence was calculated among those living in specified level of malaria intervention utilization.* | | | | | | |
